# Koch’s Experiments for the Eradication of the Cholera Bacillus

**Published:** 1885-06

**Authors:** Geo. W. Lewis

**Affiliations:** Leipzig


### Koch's Experiments for the Eradication of the Cholera Bacillus.

By Geo. W. Lewis, Jr., A. B.

In the March number of the *Medical and Surgical Journal* I endeavored to present the characteristic features of a ten days' course, under Dr. Robert Koch, on the cultivation and detection of the comma bacillus of Asiatic cholera. Several methods for artificially producing the organism were described, and some of its more salient peculiarities, in contra-distinction to others of its class, were pointed out. No mention, however, was made of the effect which certain well-known re-agents have upon its growth and activity ; and I desire, therefore, in this paper to give the results of the latest experiments which have been made with a view of finding some means for its effectual eradication. I shall also present a review of the sanitary precautions, which, under the sanction of Dr. Koch, the German government is prepared to enforce at any moment, in the event of a cholera visitation. They seem to me to form as complete a code of rules under which to act as it is possible to formulate from our present limited understanding of the organism and its life history.

Within the past year or two scarcely a week has passed but what some original worker has demonstrated before a medical society the existence of what he supposed to be a hitherto unknown organism. As a result it now requires a good-sized book to name and classify the new discoveries. For the most part these discoveries have been made in Germany, where, at present, I am sorry to say, by far the most original work in all departments of medicine is being performed. Not a few of the newly found bacteria are claimed to be pathogenic in their action upon the human system, and it is, of course, to these that the most attention is directed.

When we consider how difficult it is to distinguish between organisms which, for the mere delineation of their form, require a magnifying power of from 1,000 to 2,000 diameters, it will not seem so strange, when I say that cultivation experiments and the application of various re-agents have proved, beyond a doubt, that a large number of the so-called pathogenic bacteria are identical with others already classified. This has placed a host of would-be experimenters on their guard, and now-a-days, a new bacterium, whether pathogenic or otherwise, is no sooner fairly launched, than this army proceed to attack it from all sides. As a general rule it requires but a short time to settle its right to originality, for in such hands as these and with such tests, any striking similarity which may exist between it and another is sure to be revealed. The whole field of bacteriology, so far, at least, as it bears upon disease, is therefore placed upon a foundation which entitles it to be regarded as a most important branch in a medical education, and to my mind it is only a question of time when the intimate relation which has already been shown to exist between pathology and bacteriology, will practically merge the one into the other.

In the case of most pathogenic bacteria, some re-agent has been discovered, which, in a weaker or stronger solution, exerts a marked influence, not only on the development and activity of the existing organisms, but also on the tissues in their immediate vicinity, so that a further growth of bacteria is greatly retarded. So far as is known there are no two species of micro-organisms which are affected by the same re-agent in exactly the same manner. There has always been found to be some distinguishing effect produced in the one which is entirely or almost entirely absent in the other. Indeed, the application of re-agents has been carried to such an extent, in the endeavor to place the germ theory of disease upon a sound basis, that it is now recognized as about the only method by which the existence of a new organism can be definitely established.

The comma bacillus, from the time it was first demonstrated by Dr. Koch down to the present, has, naturally enough, been a favorite subject for discussion, not only among those capable of treating it in an intelligent manner, but also, and more particularly, and again, I suppose, naturally enough, among those utterly ignorant of even the barest rudimentary knowledge of the science which it represents. As is generally the case, the latter element, always the larger of the two, has, for the time being, quite as much influence upon the majority as the former; their assertions are, in fact, even stronger and more apt to convince than those which result from careful experiment. It is, however, to this latter class, to the one which has established its every statement through a series of reliable tests, that we are glad to turn for positive information. Let us see, therefore, what has been and is still being done in the laboratory by Dr. Koch and his colleagues in their efforts to meet if possible the next visitation with a remedy.

Iodine, whose value as an intense poison for all bacteria has long been established, was naturally the first re-agent resorted to. When employed in quantities sufficient to destroy, or, at least, assist the development of other bacteria, its action upon the comma bacillus was not perceptible; and for this reason the poisonous property which the iodine possesses is only shown when it is applied to strongly diluted masses of bacteria, as, for example, very thin anthrax-blood. Even then it is necessary that the blood containing the bacilli should be almost pure water, in order to prevent the iodine from combining with the alkalies. We are never obliged to arrest the development of infectious matters in pure water, but in the alkaline contents of the intestines, or in the blood or in the juices of the tissues; and here the iodine does not remain free, but combines at once with the alkalies. To test the influence, therefore, of this re-agent upon the comma bacillus, a watery solution of iodine was added to meat-broth which contained the minimum amount of nutriment essential to keep the organisms alive. Iodine dissolves in water in the proportion of 1 to 4,000. One cubic centimeter of the iodine-water was mixed with ten cubic centimeters of meat-broth, but the growth of the bacilli was not hindered in the least. The amount of iodine, therefore, necessary to arrest their development lies considerably above that used in this experiment; and it must be remembered that arrest of development does not imply disinfection. In fact, all the experiments which have thus far been made upon this point have aimed merely at ascertaining that amount of a substance which is requisite to hinder the growth of the bacteria; but with this the bacteria are by no means killed as should be done in disinfection. To increase this amount of iodine, however, seemed useless, for in practice larger quantities than this cannot be given.

Alcohol arrests development only when one part is added to ten parts of the nutritive fluid, *i. e.*, in the proportion of ten per cent. This concentration, however, cannot be practically utilized.

Common table salt was used in the proportion of two per cent. without influencing the growth of the bacilli in the least.

In experimenting upon artificial cultivations of comma bacilli, two facts have been ascertained: 1st, that the bacilli requires a considerable amount of nutritive substance in which to barely exist; and, 2d, this nutritive substance must not be acid. With regard to the first, the amount is rather above that essential to a luxuriant growth of most other bacteria; as to the second, experiments indicate that if either the gelatine or the meat-broth, when used as nourishing media, has the slightest acid reaction, the growth is greatly impeded, while, if the acid reaction is at all marked, the development ceases entirely. It is at the same time noteworthy that it is not all acids that seem to be unfavorable to the comma bacillus; for the cut surface of a boiled potato is known to have an acid reaction (malic acid), and yet a most luxuriant growth can be obtained from such a cultivation. So far, however, as is known, this is the only acid which is not antagonistic to its development.

In time of cholera, sulphate of iron has been considered valuable for purposes of disinfection. This substance was also tested to find what concentration was necessary to stop the development of comma bacilli. A two per cent. solution seemed to act as a preventive to growth, but did not kill the organisms outright. The property which this substance has of arresting the development of the bacilli may be explained as follows:

The nutritive solution contains a certain amount of albuminates and peptone, which serve as food for the bacteria. By adding a two per cent. solution of sulphate of iron, an abundant precipitate is formed in the nutritive medium, and the albuminates and peptone are consequently driven out. It has been thought also that the acid reaction, which makes its appearence, has a checking influence on the growth, and, indeed, this would seem to be in perfect accordance with the known effect which acids in general have upon the comma bacillus. With regard to sulphate of iron as a disinfectant, however, there appears to be little doubt that it is useless. In fact, it is reasonable to suppose that, by its use, exactly the opposite result from that intended might be obtained; for example, if a cesspool, in which comma bacilli were present, had to be disinfected, the process of putrefaction which goes on of itself is sufficient to kill the comma bacilli. Now, if sulphate of iron is added until an acid reaction is obtained, the only object gained would be the cessation of the growth of the bacteria and of the comma bacilli. The bacteria, however, are by no means killed, and the comma bacilli by this method escape from the influence of the putrefaction bacteria, which are injurious to them and are preserved instead of being destroyed. It is evident, therefore, that great care must be taken in the selection of substances for disinfection, not to employ one which will merely arrest putrefaction, but one which will effectually kill all bacteria. By using the former it is probable that the end which we are seeking to attain will be defeated, namely, the infectious matter will be preserved.

Sulphate of copper, in the proportion of 1 to 2,500, has a very powerful effect upon comma bacilli; but if we were to calculate how much sulphate of copper must be given in order to check the growth of the bacilli in the intestinal canal, we would arrive at quantities which could not be given to a human being. This substance, like a sulphate of iron, even when administered in the maximum dose, is no longer in a free state by the time it reaches the intestines, and has consequently lost its power of arresting development. To be of any real service, therefore, a substance must not only reach the intestinal canal very quickly, but it must remain in a free state.

Quinine stops the growth of comma bacilli when given in the proportion of 1 to 5,000. Here we have a substance which exerts a powerful influence over the organisms, and at the same time one that can be given with safety, but it is too slow in taking effect. Another substance even more powerful than quinine is corrosive sublimate, which effectually arrests development when administered in the proportion of 1 to 100,000.

In following out these experiments, a striking peculiarity of the comma bacillus was made known, namely, that it dies very soon after being dried. This would seem to indicate that it belongs to the group of screw-shaped bacteria, which are called spirilla, rather than to the family of bacilli; for so far as is known no member of the bacillus family undergoes death by being dried; they can, in fact, be preserved for years in the dry state, and when nourishment is again furnished, their activity at once shows itself. Especially is this true of pathogenic bacteria, such as anthrax bacilli, which, we know, form spores and eventually pass into a permanent state. Other infectious substances, for example, those of small-pox and of vaccine, do not seem to be affected in the least from want of moisture, and after being deprived of it for years, are still found to retain their power of infection. Comma bacilli, however, rarely ever show any indications of life after being dried for three hours. This is a most important question in the etiology of cholera, and investigation upon this property of the bacillus appears to have been both thorough and convincing. In France, cholera dejecta and the contents of the intestines of cholera corpses were allowed to remain in a damp condition on linen, in order that the bacilli might develop under the most favorable circumstances. After short intervals, pieces of the linen were dried, to ascertain whether during these different periods any condition of permanence had been established.

In time of cholera it cannot be doubted that infection through the linen affords the only undisputed proof of the presence of an effectual infectious substance, and if this substance has a permanent state at all, it must certainly be found on cholera linen. But all experiments have thus far failed to reveal the slightest trace of anything that would lead to the belief that comma bacilli have more than a very brief existence. If now even this brief existence, which, by the way bears a most striking resemblance to the cholera-process, can be cut short by causing them to pass into a dry state, I can imagine that, in seeking a remedy, considerable advantage can be derived from this point; it will, at least, prove of great service in warding off an epidemic.

The following is a review of the sanitary precautions recommended by Dr. Koch to the Imperial German Board of Health in case the cholera visits that country during the coming summer. They will, as far as possible, be enforced by both military and police officers throughout the empire:

Cholera patients are to be removed at once from the house in which they were attacked, to large, commodious and well-ventilated quarters on the outskirts of the town or city. The other occupants are to leave the building until the process of disinfection has been thoroughly carried out by those who have this duty in charge. I will say here that representatives from every city and town in Germany have already passed through a uniform course of instruction at Berlin under Dr. Koch and his assistants.

The infectious matter, which for the most part is to be found in the dejecta, must be destroyed immediately by the use of *sublimate*. This, however, is not as safe in the hands of people unacquainted with its use as carbolic acid. On the other hand, the latter is not so quick in taking effect as the former. Therefore, when an experienced person is near, the sublimate should by all means be employed; otherwise, carbolic acid.

Carbolic acid is to be made use of in such a quantity that the dejecta and disinfectant solution, when combined, produces a mixture yielding five per cent. carbolic acid; the disinfectant solution, therefore, when prepared, must necessarily contain considerably more than five per cent. carbolic acid. It is advisable that all dejecta be placed in covered stone jars and be allowed to stand for 24 hours under the influence of the disinfectant solution.

Sublimate should be employed in a two per cent. solution (mixture one per cent.), and should be allowed to remain in contact with the dejecta for from three-quarters to one hour. It is questionable, however, on account of the formation of albuminous compounds, whether sublimate is, under all circumstances, to be relied upon as a disinfectant for cholera dejecta.

At the end of the above mentioned periods the dejecta should be buried.

It must be remembered that washing with soap only arrests development; it does not kill the organisms. All linen and bed garments are to be repeatedly saturated with sublimate and then thoroughly dried.

The attendants should be made mindful of the danger both to themselves and to those with whom they come in contact; they should be instructed in all the precautionary measures to be made use of, and above all should allow nothing to be eaten or drunk in the sick room. Their hands, after every contact, should be washed first with sublimate and then with soap. The hands, moreover, should never touch the mouth or beard.

Cholera corpses should be buried within twelve hours after death; their clothes and linen should, as far as possible, be buried with them.

Laundrymen and all others engaged in the cleansing of soiled garments should be cautioned with regard to the danger and the means of avoiding it.

When the burial takes place within a few hours after death, there is no object to be gained in exposing the corpse to the process of disinfection, for the reason that the very part, namely the inner, which it is most desirable to influence, would not be affected by the disinfectant in so short a time.

Great care must be taken not to allow garments which have been worn either by cholera patients or by those attending them, to be misplaced and thereby escape the cleansing process. Those who have the burial arrangements in charge should, after performing the slightest service, thoroughly purify their own clothes with sublimate, and, moreover, should not wear them again until they are perfectly dry.

Soiled water which results from the cleansing of garments worn by cholera patients, must not pass off through the ordinary channels until it has been thoroughly disinfected. For this purpose, large stone jars will be found the safest and most convenient receptacles.

People who attend the funeral of a cholera corpse will not be allowed to enter the building in which he died.

Worthless effects of the deceased, together with everything in the sick room which is not considered of positive value, should be at once burned.

Whatever is to be preserved should be exposed to an atmosphere of steam for not less than one hour. At the expiration of this time, the following test will show whether the articles have been thoroughly disinfected. Placed in the middle of a pan of earth, they are to be heated in an oven for one half hour, and then the whole is to be poured out upon a plate of food-gelatine. (The description of this substance will be found in the March number of this journal.) If the disinfection has proved successful, no growth will make its appearance in the nutritive medium. If, however, at the end of 24 hours small white specks are found upon the food-gelatine, there is reason to believe that the infectious matter has not all been destroyed. A microscopic examination will then decide the question as to whether or not the white specks constitute colonies of comma bacilli.

An equally good method consists in boiling the articles in a large kettle for a similar period. A piece of wooden lattice-work can be placed upon the surface of the water, and from this clothes and other effects can be suspended. The outpouring stream should reach 100° centigrade.

The effects can likewise be boiled in solutions of sublimate or carbolic acid, and afterwards be washed with soap in the usual manner.

The conveying of cholera linen from one place to another is extremely dangerous to those engaged in this work. It should, therefore, be guarded with the utmost care, and before removal should be placed in air-tight cans, so that contact with it will not be possible after it leaves the sick room.

Among those in poor circumstances are always to be found uncleanly articles, not alone of dress but of furniture and decorations as well, which, in time of cholera, are to be regarded with suspicion; such things should be saturated with the sublimate solution and then thoroughly dried. The drying, however, should, if possible, take place in an adjoining room, under the influence of free currents of air from both doors and windows, and with a very hot fire.

Floors, especially in the crevices where dirt has collected and around the corners; also bedsteads which have been occupied by cholera patients, should be kept moist with sublimate. Open fires should be kept burning day and night in the sick room. New but perfectly dry houses are strongly recommended.

A room in which a death from cholera has occurred should not be occupied again for at least six days.

As has already been stated, a microscopic examination of suspicious dejecta, or, better still, of a piece of linen upon which the dejecta rests, will enable the observer to satisfactorily diagnose the first case. The presence or absence of comma bacilli is always noticeable, and a person acquainted with the microscopic appearance of healthy dejecta cannot fail to detect the ravages of these foreign invaders. When present, they are to be found in quantities altogether out of proportion to the other intestinal bacteria, and their characteristic form and rapid movements at once stamp them as pathogenic.

With regard to the way in which the first case was caused, the physical condition previous to the attack undoubtedly has more to do with it than anything else. Statistics show that by far the largest proportion of cases occurs on Monday and Tuesdays, that is, on the days which have been preceded by excesses in eating and drinking. It is probably true that comma bacilli under certain conditions cannot pass the stomach, at least of animals. This agrees perfectly with all our experience of cholera; for predisposition has always seemed to play an important part in cholera infection. It can be assumed that of a number of people exposed to cholera, only a fraction of them will fall ill; but, however large or small this number may be, it will generally be found that they have been suffering from some digestive disturbance. Catarrh of the stomach or intestines, or the overloading of the stomach with indigestible food, are common complaints, but are most dangerous in time of cholera. More or less undigested masses of food may pass into the intestinal canal, and bring with them the comma bacilli, which, on account of the poor work of digestion, were not killed in their passage through the stomach.

For these reasons the following precautionary measures should be taken: Water for either washing or drinking purposes should not be used unless it has been boiled for at least half an hour. If it is allowed to stand until it becomes cold, it should not be used again without being reboiled. Green vegetables, salads and all fruits shall not be eaten. Milk should be avoided as far as possible unless boiled. Great care must be taken to rinse vegetables with nothing but boiling water. All food should be throughly cooked. Milk should not be accessible to flies.

Sinks should be kept wet with sublimate and should not be used while the disease prevails. Dirty brooms and rags should either be destroyed or be kept saturated with sublimate. Disinfection of the contents of water closets is not only impossible, but, according to Dr. Koch, is of secondary importance: The seats, however, should be kept moist with sublimate.

Rivers and springs should be carefully guarded during a cholera visitation, so as not to allow the people living in their vicinity to use the water for washing or household purposes; for flowing water, especially when it drains a densily populated country, is extremely dangerous.

LEIPZIG, May 14, 1885.

